# Identification of Necrophagous Beetles (Coleoptera) Using Low-Resolution Real-Time PCR in the Buffer Zone of Kampinos National Park

**DOI:** 10.3390/insects16020215

**Published:** 2025-02-15

**Authors:** Tadeusz Malewski, Katarzyna Leszczyńska, Katarzyna Daria Borzuchowska, Maciej Sierakowski, Tomasz Oszako, Justyna Anna Nowakowska

**Affiliations:** 1 Department of Molecular and Biometric Techniques, Museum and Institute of Zoology, Polish Academy of Sciences, 00-818 Warsaw, Poland; 2 Marrow Donor Center with HLA Laboratory, Military Institute of Medicine—National Research Institute, 04-141 Warsaw, Poland; kasia.leszczynska97@gmail.com; 3 NeuroProtect Medical Center, Clinical Research in Neurological Diseases, 01-684 Warsaw, Poland; katarzyna.daria.borzuchowska@gmail.com; 4 Faculty of Christian Philosophy, Institute of Biological Sciences, Cardinal Stefan Wyszyński University in Warsaw, 01-938 Warsaw, Poland; m.sierakowski@uksw.edu.pl; 5 Forest Protection Department, Forest Research Institute, 05-090 Sekocin Stary, Poland; t.oszako@ibles.waw.pl; 6 Faculty of Biology and Environmental Sciences, Institute of Biological Sciences, Cardinal Stefan Wyszyński University in Warsaw, 01-938 Warsaw, Poland; j.nowakowska@uksw.edu.pl

**Keywords:** forensic entomology, necrophagous Coleoptera, species identification, COI, low-resolution melting real-time PCR, Kampinos National Park

## Abstract

Forensic entomology is concerned with the study of arthropods that assist in criminal investigations. Among them, necrophagous beetles (Coleoptera) play a crucial role as they are found in all stages of decomposition of corpses, which makes them valuable for forensic analysis. Accurate species identification is crucial for the effective use of entomological evidence. Efficient, cost-effective, and rapid methods are essential for this purpose. In the present study, we developed a novel assay that enables simple, inexpensive, and rapid identification of necrophagous beetle species. Using two primer sets targeting the cytochrome c oxidase 1 (COI) gene and analyzing the resulting SYBR Green I melting curves, we were able to identify fourteen beetle species: *Aleochara curtula* (Goeze, 1777), *Anoplotrupes stercorosus* (Scriba, 1791), *Creophilus maxillosus* (Linnaeus, 1758), *Dermestes undulatus* (Brahm, 1790), *Hister unicolor* (Linnaeus, 1758), *Margarinotus brunneus* (Fabricius, 1775), *Necrodes littoralis* (Linnaeus, 1758), *Nicrophorus investigator* (Zetterstedt, 1824), *Ontholestes murinus* (Linnaeus, 1758), *Oiceoptoma thoracicum* (Linnaeus, 1758), *Philonthus cognatus* (Stephens, 1832), *Saprinus planiusculus* (Motschulsky, 1849), *Silpha tristis* (Illiger, 1798), and *Thanatophilus rugosus* (Linnaeus, 1758). Data on the melting temperature of the amplicon can be obtained within 60–90 min after DNA extraction. This study expands the understanding of the species composition of necrophagous Coleoptera, which is crucial for forensic entomology methods based on insect succession. Furthermore, it provides the first comprehensive data on necrophagous beetles in the vicinity of Kampinos National Park.

## Simple Summary

Forensic entomology is the study of insects and other arthropods that help solve crimes. A key group in this field is necrophagous beetles, which are attracted to decaying corpses and can be found at every stage of decomposition. Accurate identification of beetle species is essential to utilize this evidence effectively, which requires rapid and cost-effective methods. In this study, the researchers developed a new, simple, and affordable test for the rapid identification of necrophagous beetle species. This method determines the melting temperature of the cytochrome c oxidase 1 gene amplicon, which is sequence-dependent and enables species identification. This approach enabled the identification of 14 species of beetles, including some found near Kampinos National Park in Poland, representing the first comprehensive study in this area. The entire process, from DNA extraction to species identification, takes only 60–90 min. This study expands the understanding of the species composition of necrophagous Coleoptera, which is crucial for forensic entomology methods based on insect succession. Furthermore, it provides the first comprehensive data on necrophagous beetles in the vicinity of Kampinos National Park.

## 1. Introduction

The estimation of the postmortem interval (PMI) plays a pivotal role in forensic investigations to aid in death investigations. Initially, the predictable physical and chemical consequences of death are usually the most reliable PMI indicators, but as the time since death increases, the above methods become less useful, and more accurate results are often obtained using entomological information [1]. Insects are attracted to decaying remains within hours of death, with their colonization and development providing a wealth of information for forensic analysis. The accuracy of insect-based PMI estimates depends on many factors, such as the correct identification of the species present on the corpse. As ectothermic organisms, insects’ growth and development rates are temperature-dependent, a property utilized in the development method of PMI estimation. This method relies primarily on the immature stages of blowflies, particularly those of the family Calliphoridae, whose life cycles are well documented. While Calliphoridae provide reliable PMI estimates for the early stages of decomposition, their utility is limited to the larval stage of their life cycle [1,2,3,4].

Necrophagous beetles (Coleoptera) are valuable in addressing this gap, as they usually colonize remains across all decomposition stages [5]. Certain genera, such as *Thanatophilus* (Leach, 1815) and *Necrodes* (Leach, 1815), colonize remains within hours of death, and many species have a relatively slow rate of development, which increases their usefulness for PMI determination. For example, *Thanatophilus* species are widespread and often colonize corpses within 24 h post-mortem, making them suitable for PMI estimation [6]. In woodland and desert environments, *Dermestes maculatus* (De Geer, 1774) colonization can occur even prior to calliphorids [7]. Most families, however, are associated with later stages of decomposition or skeletal remains [8,9]. By incorporating beetle data, forensic entomologists can expand the window for PMI estimation and improve accuracy in cases of advanced decomposition.

PMI can also be estimated using known insect succession patterns on cadavers. The composition and abundance of insect species on cadavers can vary depending on habitat type, climate, and other factors, so this method requires knowledge of the composition of the local carrion fauna [10,11].

Despite their forensic significance, identifying necrophagous beetle species—especially in the immature stage—remains challenging, with DNA barcoding offering a solution. DNA barcoding is effective in identifying species at all stages of development; however, it is rather time-consuming and resource-intensive, limiting its application in large-scale investigations [12].

Real-time PCR coupled with melting curve analysis offers a promising alternative for species identification, allowing for differentiation based on different melting temperatures of the PCR products. High-resolution melting PCR (HRM-PCR) provides very detailed data and is, therefore, suitable for the identification of a wide range of organisms, including bacteria, insects, fish, and mammals, e.g., [13,14,15,16]. However, the application of HRM-PCR is limited by its requirement for specialized equipment. Alternatively, low-resolution melting PCR (LRM-PCR) offers a more accessible approach, as it can be performed using most standard real-time PCR thermocyclers. LRM-PCR has demonstrated effectiveness in various fields, including meat authentication, immunology, virology, and the detection of pathogens in food and water [17,18,19].

Kampinos National Park (KPN), encompassing an area of 37,756 hectares, is the second-largest protected area in Poland. Its diverse habitats—comprising grasslands, dunes, pine forests, meadows, fields, and pastures—support a remarkable variety of beetle species [20,21]. Despite its biological richness, KPN remains one of the most valuable and, at the same time, least-known national parks in Poland in terms of entomological research [22]. It is worth noting that the proximity of KPN to Warsaw, approximately 40 km away, adds practical significance. Knowledge of the species composition of insects may provide valuable insights for law enforcement agencies, given the higher crime rates associated with large urban areas like Warsaw.

In this study, we evaluated the utility of low-resolution melting PCR (LRM-PCR) as the rapid and reliable method for identifying necrophagous beetle species. The developed assay, utilizing two primer sets targeting the COI gene, combined with LRM-PCR, successfully identified half of the beetle species collected within the buffer zone of Kampinos National Park (KNP). This research highlights the potential of LRM-PCR as a tool for forensic entomology while providing the first dataset on necrophagous beetles in the KNP buffer zone, contributing valuable ecological knowledge.

## 2. Materials and Methods

### 2.1. Sampling and Generation of the Standard COI Barcode

Specimens of necrophagous Coleoptera were collected between April and September 2014 from pig carcasses in the buffer zone of Kampinos National Park 40 km north of Warsaw (52°21′ N; 20°47′ E). Three domestic pig carcasses were obtained from a local slaughterhouse. The study site was a meadow adjacent to a forest area, where single pig carcasses were placed in cages at the beginning of April, June, and August 2014. Six traps were placed 40 cm apart around the cage, each containing a small hole dug in the ground and a vial. Additionally, beetles present on and in the carcasses and on the soil were collected manually. Sampling was conducted during 60-day periods. During this period, the carcasses were completely decomposed. During the first two weeks, carcasses were examined every day and afterward, every week. In total, about 300 beetle specimens were collected. Genomic DNA was extracted from the legs or whole bodies of the small beetles using a GeneMATRIX Tissue DNA Purification Kit (EURX, Gdańsk, Poland) according to the kit’s instructions. The extracted DNA concentration was normalized to 25 ng/mL and stored at −20 °C.

The COI barcode region was amplified with a combination of primers: LCO1480 (GGTCWACWAATCATAAAGATATTGG) and HCO2198 (TAAACTTCAGGGTGACCAAARAAYCA) [23]. PCR amplification of the COI barcode region was performed in 40 μL reaction volume containing 20 μL REDTaq Ready Mix (Merck, Darmstadt, Germany), 4 μL 5 μM forward and reverse primers, 2 μL DNA extract, and H_2_O to 40 μL. Thermal cycling was performed on a Veriti 96-well thermal cycler (Applied Biosystems, Foster City, CA, USA) with the following program: an initial denaturation step at 94 °C for 5 min, followed by 35 cycles at 95 °C for 30 s, 61 °C for 30 s, 72 °C for 30 s, and a final extension step at 72 °C for 10 min. Excess dNTPs and unincorporated primers were removed using a Clean-Up Purification Kit (A&A Biotechnology, Gdynia, Poland). The purified DNA was eluted in 40 μL sterile H_2_O. Sequencing PCR reactions were prepared in 10 μL volumes and contained 1 μL BigDye Terminator, 2 μL 5× sequencing buffer (BigDye Terminator v3.1 Cycle Sequencing Kit, Applied Biosystems, Waltham, MA, USA), 1.6 μL (10 μM) forward or reverse mini barcode primer, and 1.5 μL DNA and H2O to 10 μL total volume. The thermal profile for the sequencing reactions consisted of 25 cycles at 96 °C for 1 min, 96 °C for 10 s, 50 °C for 5 s, and 60 °C for 1.45 min. Amplicons were sequenced using a 3500 xL Genetic Analyzer (Applied Biosystems, Foster City, CA, USA) [15].

### 2.2. Primer Design for the Amplicon Melting Curve Analysis

The COI sequences obtained were aligned with Clustal W [24]. Areas with high sequence similarity were identified for the design of universal primers. Primer design was performed using Primer3 software, v.4.1.0 [25], and primers were synthesized by Merck (Darmstadt, Germany). PCR reactions for melting curve analysis were performed using a BioRad CFX96 thermal cycler (Bio-Rad, Hercules, CA, USA). EvaGreen (Biotium, Fremont, CA, USA) loading dye was used to prevent dye redistribution during DNA melting and to obtain a more accurate measurement of the amplicon melting temperature in the reaction. Each 20 μL reaction mixture contained 10 μL Real Time 2 × RT PCR Mix EvaGreen (A&A Biotechnology, Gdynia, Poland), 2 μL 5 μM of each primer, 2 μL (50 ng) DNA extract, and 4 μL H_2_O to a total volume of 20 μL. To maximize reproducibility, the same amount of DNA (50 ng) was used for amplification, and samples were not added to the outer wells of the plate. The target sequences were amplified using a thermal program with an initial denaturation step of 95 °C for 3 min, followed by 40 cycles of 95 °C for 10 s, 61 °C for 30 s, and 72 °C for 15 s. The melting curve was determined by observation. The melting curve was analyzed by observing the fluorescence changes when the temperature was increased from 65 °C to 95 °C.

## 3. Results

### 3.1. Preparation of Reference DNA Samples

The amplified COI products were sequenced using the Sanger sequencing method. The resulting sequences were deposited in GenBank and identified at the species level by BLASTn analysis. The sequences obtained from 94 specimens were assigned to 29 Coleoptera species (Table S1). The degree of identity of these sequences, except for *Creophilus maxillosus* (Linnaeus, 1758) (PQ740169), ranged from 97.03% [*Ontholestes murinus* (Linnaeus, 1758), PQ740150] to 100% [*Phelotrupes auratus* (Motschulsky, 1857), PQ740106; *Anisotoma glabra* (Kugelann, 1794) PQ740105; *Catops fuscus* (Müller, 1764) PQ740101)].

The identified species were distributed across twelve Coleoptera families, with two families being the most species-rich. The Silphidae family comprised six species: *Necrodes littoralis* (Linnaeus, 1758), *Nicrophorus investigator* (Zetterstedt, 1824), *Oiceoptoma thoracicum* (Linnaeus, 1758), *Silpha tristis* (Illiger, 1798), *Thanatophilus rugosus* (Linnaeus, 1758), and *Thanatophilus sinuatus* (Fabricius, 1775). The Histeridae family included five species: *Hister unicolor* (Linnaeus, 1758), *Hypocaccus rugifrons* (Paykull, 1798), *Margarinotus brunneus* (Fabricius, 1775), *Saprinus planiusculus* (Motschulsky, 1849), and *Saprinus semistriatus* (Scriba, L. G., 1790). The Staphylinidae were represented by four species: *Aleochara curtula* (Goeze, 1777), *C. maxillosus*, *O. murinus*, and *Philonthus cognatus* (Stephens, 1832). The last nine families comprised 1–3 species.

### 3.2. Analysis of the DNA Melting Profile

**Figure 1:**
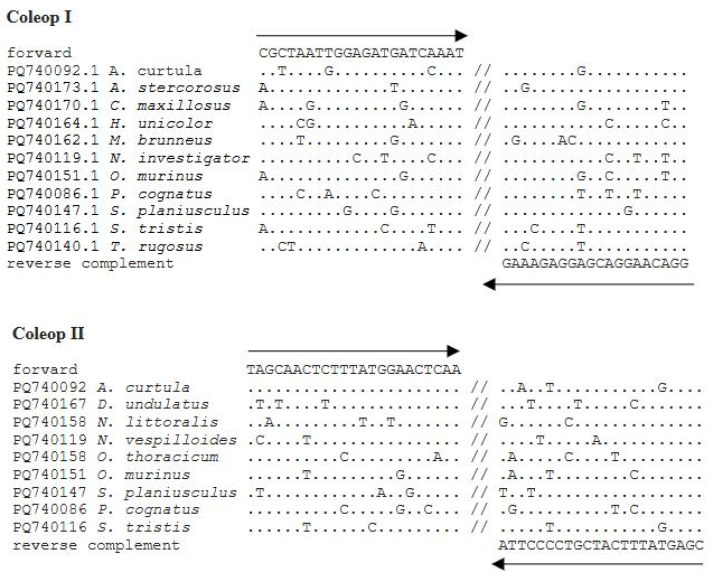
Similarity of the COI gene sequences of the studied species to the designed primers. Identical nucleotides are given as dots.

The COI sequences obtained were aligned; conserved regions were identified, and two primer pairs were designed: Coleop I (forward: CGCTAATTGGAGATGATCAAAT and reverse: CCTGTTCCTGCTCCTCTTTC) and Coleop II (forward: TAGCAACTCTTTATGGAACTCAA and reverse: GCTCATAAAGTAGCAGGGGAAT) The sequence similarity to the designed primers is shown in Figure 1.

The Coleop I primers amplify COI in eleven Coleoptera species, while the Coleop II primers amplify COI in nine species. Coleop I fails to amplify COI in *D. undulatus* (Brahm, 1790), *N. littoralis*, and *O. thoracicum*, while Coleop II fails in *Anoplotrupes stercorosus* (Scriba, 1791), *C. maxillosus*, *H. unicolor*, *M. brunneus*, and *T. rugosus*. Both primer pairs successfully amplify COI in six species: *A. curtula*, *Nicrophorus vespilloides* (Zetterstedt, 1824), *O. murinus*, *P. cognatus*, *S. planiusculus*, and *S. tristis*).

The melting temperature (Tm) of the Coleop I amplicons ranged from 72.1 °C to 78.0 °C. The Tm values of Coleop I for *S. planiusculus* and *A. curtula* were almost identical (76.0 °C and 76.1 °C, respectively), but the Tm of the Coleop II amplicon differed significantly between these species (*S. planiusculus*: 69.4 ± 0.16 °C, *A. curtula*: 70.0 ± 0.10 °C). Similarly, the Coleop II amplicons of *A. curtula* and *S. tristis* exhibited identical Tm values (70.0 °C), while the Coleop I amplicons of these species displayed significant differences in Tm (*A. curtula*: 76.1 ± 0.16 °C, *S. tristis*: 76.5 ± 0.11 °C) (Table 1).

**Figure 2:**
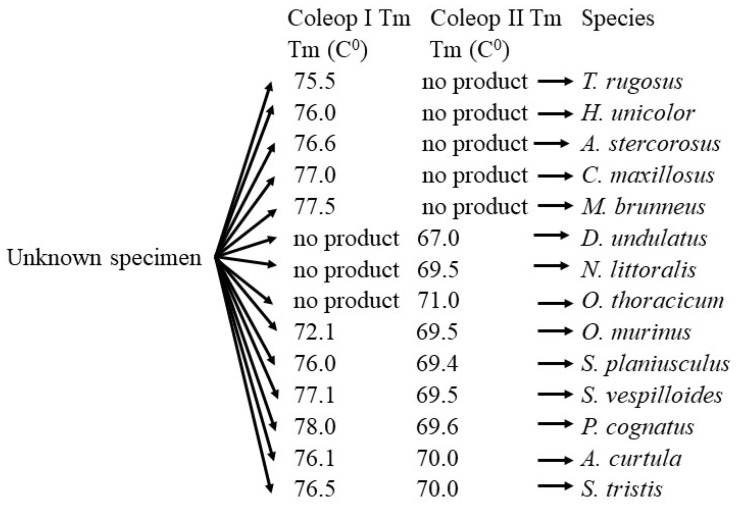
The workflow for the identification of necrophagous Carabidae.

Based on the data obtained, a workflow for the identification of necrophagous Carabidae is proposed (Figure 2).

## 4. Discussion

The application of entomological methods for estimating the postmortem interval (PMI) has gained increasing recognition over the past decade. The introduction of molecular techniques, such as DNA barcoding, has addressed many of these challenges by enabling accurate species identification at all developmental stages [26,27,28]. However, the time and cost associated with barcoding limit its application in forensic applications.

Among various molecular methods for species identification, high-resolution melting (HRM-PCR) and low-resolution melting (LRM-PCR) stand out as promising approaches. These methods enable the rapid, single-tube assignment of an analyzed sample to a species based on its amplicon melting profile. The melting temperature (Tm) is influenced by both the length and GC content of the amplicon, as guanine and cytosine are bound by three hydrogen bonds, whereas adenine and thymine are bound by two. Consequently, amplicons with high GC content exhibit higher Tm values compared to those with lower GC content. In this study, we utilized the EvaGreen dye to measure Tm, as this fluorescent dye does not redistribute during DNA melting, thereby enhancing the accuracy of Tm measurements [29].

This study demonstrated the potential of LRM-PCR as a practical alternative for the rapid and cost-effective identification of necrophagous beetles. By using Tm profiles derived from the cytochrome c oxidase I (COI) gene, we identified 14 beetle species (Table 1). Our findings align with previous studies where LRM-PCR has been successfully applied to sex determination [30], the detection of heterozygosity at the HUMTHO1 locus [31], and the identification of single nucleotide polymorphisms (SNPs) [32]. The test is economical and rapid. It requires only a set of primers and a saturating fluorescent dye and takes approximately two hours to perform. Real-time PCR combined with species-specific probes offers a simple and time-efficient approach. However, it is associated with higher costs, and its capacity to identify species is constrained by the limited number of detection channels available on the instruments [17]. HRM-PCR utilizes saturated melting dyes, which are cost-effective and have been successfully applied in forensic entomology [14,15,33,34,35,36] but require thermal cyclers with high-temperature uniformity. Currently, only a few companies (Bio Molecular Systems, London, UK; Qiagen, Venlo, the Netherlands; Roche, Basel, Switzerland) have produced this type of qPCR cycler.

The presence of specific insect species or their larvae on human remains can offer critical insights into the circumstances surrounding a death, such as the location of the body, potential trauma, or postmortem movement. The insect fauna collected from such studies contributes to the enrichment of datasets, providing valuable information that can be extrapolated and applied to regions with similar landscapes or climatic conditions.

Carrion, or dead animal material, provides a temporary and variable food source for a diverse and varied community of organisms [37]. Insects, especially Coleoptera and Diptera, are the main components of this community and represent a major element of the decomposition process. The order Coleoptera includes a number of forensically important families: Staphylinidae, Nitidulidae, Scarabaeidae, Silphidae, Dermestidae, and Histeridae [1]. The carrion beetles (Silphidae) belong to a group of insects that are closely associated with the decomposition of animal remains and other decaying organic materials. The family Silphidae is divided into two subfamilies: Silphinae and Nicrophorinae. Worldwide, this family comprises 183 species spread over 15 genera. In Northwestern Europe, 28 species have been recorded: 17 from the subfamily Silphinae and 11 from the subfamily Nicrophorinae. Although the Silphidae are distributed worldwide, they are most common in temperate regions. Most species of the Silphidae family are scavengers that feed on decaying matter, but they also feed on other organisms associated with carrion, such as fly eggs, maggots, and smaller carrion beetles [38,39]. Six species of Silphidae were observed in this study (Table S1). When examining the faunal sequence of beetles on the carcasses, carrion beetles (Silphidae) are the first to be attracted, followed by weevils (Staphylinidae) and clown beetles (Histeridae) [38]. Among them, *Necrodes littoralis* stands out in Central Europe due to its frequent visits and broods on large vertebrate carcasses, especially in spring and summer. This species colonizes carcasses in the later stages of decomposition, significantly extending the period during which the post-mortem interval (PMI) can be estimated from insect activity. More than 90% of the documented cases occurred outdoors, especially in forests, bushes, and fields [40]. Other important necrophagous species are *Thanatophilus sinuatus* and *T. rugosus*. These beetles are broadly distributed across the Palaearctic region and are commonly found on both human and animal cadavers [41]. The second largest species-rich family is the Histeridae. Beetles of the family Histeridae are a stable component of carrion and dung communities. Both adult and immature Histeridae are typically found in association with decaying animal or plant matter, suggesting that they are primarily scavengers [42]. In our study, we also identified five species of Histeridae (Table S1). *Saprinus* spp. is usually abundant on decaying carcasses and occurs in a variety of habitats. Their occurrence and presence are related to bloating and active decomposition. Temperature and food availability appear to be the most important factors for the presence of *S. semistriatus* [43]. While many of the detected species (Table S1), in particular *M. brunneus* (Histeridae), *C. maxillosus* (Staphylinidae), *Saprinus detersus* (Illiger, 1807) (Histeridae), and *Thanatophilus sinuatus* (Silphidae), have been associated with the putrefaction stages of cadaver decomposition, *Dermestes frischii* (Kugelann, 1792) and *Dermestes undulatus* are most commonly found at outdoor and indoor crime scenes during the dry and skeletal stages of decomposition [44]. Eight species of the genus Dermestes have been identified in Europe: *D. frischii*, *D. maculatus*, *D. undulatus*, *D. ater* (DeGeer, 1774), *D. bicolor* (Fabricius, 1781), *D. haemorrhoidalis* (Küster, 1852), *D. lardarius*, and *D. peruvianus*. In arid environments, Dermestes species are probably the only necrophagous insects that feed on decaying remains [45,46].

Comprehensive surveys of the necrophagous Coleoptera of Poland in the Wielkopolskie Voivodeship in central Poland have revealed the presence of beetles of the families Silphidae [47], Staphylinidae [48,49], and Dermestidae [50]. During investigations on a military training area near Poznań, it was found that in addition to the Silphidae, representatives of the Geotrupidae and Histeridae also belonged to the dominant necrophagous beetle communities. At the same time, adult individuals of *Anoplotrupes stercorosus* and *Hydrotaea similis* (Meade, 1887) reached minimum abundance on all pig carcasses in all seasons [51]. In Western Poland, pine-oak, hornbeam-oak, and alder forests had a similar composition of carrion beetles, including adults of *N. littoralis*, *T. rugosus*, *C. maxillosus*, *Omalium rivulare* (Paykull, 1789), *Oxypoda acuminata* (Hochhuth, 1860), and *Philonthus* spp., as well as larvae of *N. littoralis*, *C. maxillosus,* and *Philonthus* spp. However, differences were observed in the occurrence and activity times of certain taxa in the different forest types [51]. Similarly, fluctuations in the seasonal activity of Silphidae were described by Urbański and Baraniak [52]. The beetles from the family Silphidae were also frequently found on decaying pig carcasses in the forests of Subcarpathian Voivodeship (Southeast Poland) [53] and in the Masurian Lake District in Northeast Poland [54]. The occurrence of necrophages from the Staphylinidae family has been reported all over Poland [2,49,55]. In addition to the Wielkopolskie Dermestidae (*Dermestes haemorrhoidalis*), mummified human remains were collected from a dwelling in the Lower Silesian Voivodeship, i.e., Southwest Poland [56].

In Kampinos National Park and its buffer area, Coleoptera studies were limited to groups of beetles living in decaying birch wood [57] coprophagous and represented by 33 species belonging to three families: Geotrupidae, Hydrophilidae, and Scarabaeidae [58]; ladybird beetles [59], Scarabaeidae [60,61], Tetratomidae [62], and Ptiliidae [63]; and some families of Cleroidea [37,64].

This study adds to the growing body of the literature on necrophagous beetles in Poland and provides the first comprehensive dataset from the buffer zone of Kampinos National Park, where such data were previously lacking. These data are important not only for forensic entomology but also for ecological research. Our data align with previous surveys conducted in various Polish regions, such as Wielkopolska and Subcarpathian Voivodeship, where beetle communities exhibited distinct seasonal and habitat-specific patterns.

## 5. Conclusions

Current advanced molecular methods such as DNA barcoding or HRM-PCR allow for precise identification of beetle species in both the adult and immature stages but require specialized laboratories. The currently developed diagnostic key based on amplicon melting profiles requires relatively simple real-time PCR thermocyclers, allowing a broader application of molecular techniques in forensic entomology. Forensic entomology studies based on insect succession can particularly benefit from this approach because they require the analysis of many samples. Both forensic entomology and faunistic research can benefit from the future development of diagnostic keys for species identification based on simple methods.

## Figures and Tables

**Table 1:** Melting temperature (Tm) of the COI amplicons of Coleop I and Coleop II. The Tm represents the mean value of three replicates.

Species	Amplicon Tm (°C). Mean ± SD	
	Coleop I	Coleop II
*Dermestes undulatus*	no product	67.0 ± 0.17
*Necrodes littoralis*	no product	69.5 ± 0.11
*Oiceoptoma thoracicum*	no product	70.1 ± 0.14
*Thanatophilus rugosus*	75.5 ± 0.11	no product
*Hister unicolor*	76.0 ± 0.14	no product
*Anoplotrupes stercorosus*	76.6 ± 0.15	no product
*Creophilus maxillosus*	77.0 ± 0.13	no product
*Margarinotus brunneus*	77.5 ± 0.11	no product
*Ontholestes murinus*	72.1 ± 0.10	69.5 ± 0.12
*Saprinus planiusculus*	76.0 ± 0.09	69.4 ± 0.16
*Nicrophorus vespilloides*	77.1 ± 0.17	69.5 ± 0.11
*Philonthus cognatus*	78.0 ± 0.15	69.6 ± 0.09
*Aleochara curtula*	76.1 ± 0.16	70.0 ± 0.10
*Silpha tristis*	76.5 ± 0.11	70.0 ± 0.14

## Data Availability

The sequence data presented in this study were deposited in NCBI GenBank.

## Supplementary Materials

The following supporting information can be downloaded at https://www.mdpi.com/article/10.3390/insects16020215/s1, Table S1: Minimal identity of COI sequence of the specimens.
