# Supplementary material for: Identification of Necrophagous Beetles (Coleoptera) Using Low-Resolution Real-Time PCR in the Buffer Zone of Kampinos National Park
Source: Insects. 2025 Feb 15;16(2):215. doi: 10.3390/insects16020215 (PMC11856018; doi:10.3390/insects16020215)
Supplement: Supplementary file 1 [file insects-16-00215-s001.zip › insects-3428817-supplementary.pdf]

Table S1. Minimal identity of COI sequence of the specimens.

Identity (%)	GenBank Acc no	BLAST top-hit	Specimen GenBank Acc no
		Closest Match	
98.94	KU906252.1	*Aleochara curtula* (Goeze, 1777)	PQ740092
98.81	KU906252.1	*A. curtula*	PQ740091
98.93	KU906252.1	*A. curtula*	PQ740090
99.24	KU906252.1	*A. curtula*	PQ740089
98.96	KU906252.1	*A. curtula*	PQ740088
99.37	KU906252.1	*A. curtula*	PQ740087
100.00	KM443099.1	*Anisotoma glabra* (Kugelann, 1794)	PQ740105
98.72	KM451400.1	*Anoplotrupes stercorosus* (Scriba, 1791)	PQ740173
99.10	KM451400.1	*A. stercorosus*	PQ740172
98.89	HQ165003.1	*A. stercorosus*	PQ740171
99.26	KF317270.1	*Athous subfuscus* (Müller, 1764)	PQ740124
99.10	KF317270.1	*A. subfuscus*	PQ740123
99.36	KF317270.1	*A. subfuscus*	PQ740122
100.00	KF317270.1	*A. subfuscus*	PQ740121
99.27	KF317270.1	*A. subfuscus*	PQ740120
97.09	HM411773.1	*Carabus nemoralis* (Müller, 1764)	PQ740137
98.97	HM411773.1	*C. nemoralis*	PQ740136
99.46	HM411773.1	*C. nemoralis*	PQ740135
99.16	KM449140.1	*Catops fuscus* (Panzer, 1794)	PQ740104
99.23	KM449140.1	*C. fuscus*	PQ740103
99.36	KM449140.1	*C. fuscus*	PQ740102
99.39	KJ966129.1	*Creophilus maxillosus* (Linnaeus, 1758)	PQ740170
96.75	KJ966129.1	*C. maxillosus*	PQ740169
99.00	KJ966129.1	*C. maxillosus*	PQ740168
97.40	KU494101.1	*Dermestes undulatus* (Brahm, 1790)	PQ740167
98.67	KU494101.1	*D. undulatus*	PQ740166
98.70	KU494101.1	*D. undulatus*	PQ740165
99.09	JX064157.1	*Geotrupes stercorarius* Linnaeus, 1758	PQ740108
99.19	JX064157.1	*G. stercorarius*	PQ740107
99.15	KU915456.1	*Hister unicolor* (Linnaeus, 1758)	PQ740164
99.44	KU915456.1	*H. unicolor*	PQ740163
98.82	KU915456.1	*H. unicolor*	PQ740160
99.20	JF889776.1	*Hypocaccus rugifrons* (Paykull, 1798)	PQ740114
99.42	JF889776.1	*H. rugifrons*	PQ740113
98.83	KU908440.1	*Margarinotus brunneus* (Fabricius, 1775)	PQ740162
98.49	KU908440.1	*M. brunneus*	PQ740161
98.97	KU908440.1	*M. brunneus*	PQ740159

99.65	MG456750.1	*Necrobia ruficollis* (Fabricius, 1775)	PQ740131
97.98	KM452149.1	*Necrodes littoralis* (Linnaeus, 1758)	PQ740158
98.52	KM452149.1	*N. littoralis*	PQ740157
98.65	KM452149.1	*N. littoralis*	PQ740156
98.09	KU915489.1	*Nicrophorus investigator* (Zetterstedt, 1824)	PQ740119
98.50	KU915489.1	*N. investigator*	PQ740118
99.36	KU915489.1	*N. investigator*	PQ740117
99.35	KU915079.1	*Nitidula rufipes* (Linnaeus, 1767)	PQ740128
99.16	KU915079.1	*N. rufipes*	PQ740127
99.85	KU915079.1	*N. rufipes*	PQ740126
99.48	KU915079.1	*N. rufipes*	PQ740125
99.01	MZ659350.1	*Oiceoptoma thoracicum* (Linnaeus, 1758)	PQ740155
98.68	MZ659350.1	*O. thoracicum*	PQ740154
98.19	MZ659350.1	*O. thoracicum*	PQ740153
98.74	LR742640.1	*O. thoracicum*	PQ740152
98.87	KM440670.1	*Ontholestes murinus* (Linnaeus, 1758)	PQ740151
97.03	KM440670.1	*O. murinus*	PQ740150
99.11	KM440670.1	*O. murinus*	PQ740149
98.92	KM444600.1	*O. murinus*	PQ740148
100.00	OL343377.1	*Phelotrupes auratus* (Motschulsky, 1857)	PQ740106
98.24	KR485683.1	*Philonthus cognatus* (Stephens, 1832)	PQ740086
98.85	KR485683.1	*P. cognatus*	PQ740085
99.32	KR485683.1	*P. cognatus*	PQ740084
99.43	KR485683.1	*P. cognatus*	PQ740083
99.37	KR485683.1	*P. cognatus*	PQ740082
99.07	KR485683.1	*P. cognatus*	PQ740081
99.01	KM441423.1	*Ptenidium nitidum* (Heer, 1841)	PQ740096
99.21	KM441423.1	*P. nitidum*	PQ740095
99.38	KM441423.1	*P. nitidum*	PQ740094
99.48	KM441423.1	*P. nitidum*	PQ740093
99.27	MN454714.1	*Pterostichus nigrita* (Paykull, 1790)	PQ740134
99.26	MN454714.1	*P. nigrita*	PQ740133
99.34	MN454714.1	*P. nigrita*	PQ740132
98.57	MH307935.1	*Saprinus planiusculus* (Motschulsky, 1849)	PQ740147
99.52	MH307935.1	*S. planiusculus*	PQ740146
99.26	MH307935.1	*S. planiusculus*	PQ740145
99.18	KM439324.1	*Saprinus semistriatus* (L.G.Scriba, 1790)	PQ740112
99.28	KM439324.1	*S. semistriatus*	PQ740111
99.16	KM439324.1	*S. semistriatus*	PQ740110
99.36	KM439324.1	*S. semistriatus*	PQ740109
99.18	KM849301.1	*Sciodrepoides watsoni* (Spence, 1813)	PQ740100

97.75	KM849301.1	*S. watsoni*	PQ740099
98.90	KM849301.1	*S. watsoni*	PQ740098
99.46	MZ609983.1	*S. watsoni*	PQ740097
98.74	HQ559261.1	*Silpha tristis* (Illiger, 1798)	PQ740116
99.25	HQ559261.1	*S. tristis*	PQ740115
99.25	HQ559261.1	*S. tristis*	PQ740174
99.04	KU916971.1	*Stephostethus lardarius* (DeGeer, 1775)	PQ740130
99.14	KU916971.1	*S. lardarius*	PQ740129
98.50	KM441510.1	*Thanatophilus sinuatus* (Fabricius, 1775)	PQ740144
98.14	KM441510.1	*T. sinuatus*	PQ740143
99.47	KM441510.1	*T. sinuatus*	PQ740142
99.05	KM441510.1	*T. sinuatus*	PQ740141
99.19	KJ963777.1	*Thanatophilus rugosus* (Linnaeus, 1758)	PQ740140
95.26	KJ963777.1	*T. rugosus*	PQ740139
99.41	KJ963777.1	*T. rugosus*	PQ740138
